# Pseudomonas stutzeri as an alternative host for membrane proteins

**DOI:** 10.1186/s12934-017-0771-0

**Published:** 2017-09-20

**Authors:** Manuel Sommer, Hao Xie, Hartmut Michel

**Affiliations:** Max Planck Institute of Biophysics, Max-von-Laue Str. 3, 60438 Frankfurt am Main, Germany

**Keywords:** Pseudomonas, Membrane protein, Overproduction, Production host

## Abstract

**Background:**

Studies on membrane proteins are often hampered by insufficient yields of the protein of interest. Several prokaryotic hosts have been tested for their applicability as production platform but still *Escherichia coli* by far is the one most commonly used. Nevertheless, it has been demonstrated that in some cases hosts other than *E. coli* are more appropriate for certain target proteins.

**Results:**

Here we have developed an expression system for the heterologous production of membrane proteins using a single plasmid-based approach. The gammaproteobacterium *Pseudomonas stutzeri* was employed as a new production host. We investigated several basic microbiological features crucial for its handling in the laboratory. The organism belonging to bio-safety level one is a close relative of the human pathogen *Pseudomonas aeruginosa*. *Pseudomonas stutzeri* is comparable to *E. coli* regarding its growth and cultivation conditions. Several effective antibiotics were identified and a protocol for plasmid transformation was established. We present a workflow including cloning of the target proteins, small-scale screening for the best production conditions and finally large-scale production in the milligram range. The GFP folding assay was used for the rapid analysis of protein folding states. In summary, out of 36 heterologous target proteins, 20 were produced at high yields. Additionally, eight transporters derived from *P. aeruginosa* could be obtained with high yields. Upscaling of protein production and purification of a Gluconate:H^+^ Symporter (GntP) family transporter (STM2913) from *Salmonella enterica* to high purity was demonstrated.

**Conclusions:**

*Pseudomonas stutzeri* is an alternative production host for membrane proteins with success rates comparable to *E. coli*. However, some proteins were produced with high yields in *P. stutzeri* but not in *E. coli* and vice versa. Therefore, *P. stutzeri* extends the spectrum of useful production hosts for membrane proteins and increases the success rate for highly produced proteins. Using the new pL2020 vector no additional cloning is required to test both hosts in parallel.

**Electronic supplementary material:**

The online version of this article (doi:10.1186/s12934-017-0771-0) contains supplementary material, which is available to authorized users.

## Background

Cells are surrounded by complex envelopes that control the exchange of metabolites, catabolites, energy and signals with the environment [1, 2]. For this manifold requirements, they rely on membrane integrated proteins that differ in structure and function. Up to 30% of all genes in eukaryotic and prokaryotic genomes encode such membrane proteins [3] and their malfunction often causes serious diseases like diabetes or cystic fibrosis [4, 5]. To gain insight into the structure and function of membrane proteins, it is crucial to purify them in sufficient amounts and in a properly folded state [6]. As they are usually not sufficiently abundant in the native cell membrane, numerous expression systems for the recombinant production of membrane proteins have been established. They differ in the host organism used, the transcriptional regulation or the post-translational modifications of the proteins [7, 8]. However, due to their hydrophobic nature, studies on membrane proteins are challenging and in many cases already their production fails.

*Escherichia coli* is the most commonly used prokaryotic host for the recombinant production of membrane protein. The organism is comprehensively characterized and can be cultivated in a cost efficient manner. Growth in rich and minimal media to high cell densities is possible over a wide range of temperatures within the course of 1 day. *Escherichia coli’s* genome was sequenced in 1997 [9] facilitating the directed design of specialized strains for protein production [10, 11]. Genetic tools to create gene deletion or insertion mutants are available and transient gene expression is enabled by the availability of a large number of vectors and promoters [12]. Taken together, *E. coli* is part of the most advanced prokaryotic production systems available but still membrane protein production is a bottleneck in many studies. Different Bacillus species, *Caulobacter crescentus*, *Pseudomonas fluorescens*, *Lactococcus lactis* and several further species are successfully used for the commercial production of predominantly soluble proteins [7], but only *L. lactis* is frequently used as an alternative host for the production of membrane proteins [13]. As a Gram-positive bacterium it differs from *E. coli* in several aspects, e.g. membrane structure or the composition of the folding and insertion machinery for membrane proteins [14]. Some of its features are thought to be beneficial for the production of proteins that could not be produced in *E. coli* and purification from *L. lactis* was demonstrated for several proteins that failed in *E. coli* [15].

Generally the choice of the appropriate host for the production of a certain protein can be crucial, but at least for membrane proteins the number of hosts to choose from is limited and many suggested candidates did not become commonly accepted at least in some cases due to their laborious handling.

In this study, we investigated the Gram-negative bacterium *Pseudomonas stutzeri* ZoBell for its suitability as a host for membrane protein production. Like *E. coli*, *P. stutzeri* belongs to the class of gammaproteobacteria and strains of the species have been isolated from aquatic and terrestrial habitats. *Pseudomonas stutzeri* has been used as a model organism to study the denitrification process [16] and bacterial diversity [17]. Recently, the species has received increasingly wide interest in biotechnological applications such as the biodegradation of environmental pollutants [18, 19]. In rare cases, strains of the species have been identified as opportunistic human pathogens but generally the species is considered as safe and can be handled in laboratories with the lowest safety level for genetic engineering work. *Pseudomonas stutzeri* grows aerobically over a wide range of temperatures in rich media or chemically defined minimal media with a sole carbon source. All strains are facultative anaerobes and use nitrate as terminal electron acceptor during anaerobic respiration [20]. Colonies are visible on agar plates after 18–24 h and cells can be kept frozen with 50% (v/v) glycerol as cryoprotectant at −80 °C for long-term storage. The first complete genome of strain *P. stutzeri* A1501 was published in 2008 [21] and since then numerous further genomes were sequenced including the strain ZoBell that was used in this study [22].

With the newly constructed broad-host-range vector pL2020 we could produce 28 out of 44 tested membrane transport proteins in *P. stutzeri* with high yields. Our results illustrate *P. stutzeri’s* capability as an alternative host for membrane protein production, further extending its possible applications in biotechnology.

## Results

### Growth characteristics of *P. stutzeri*

Bacteria used for the heterologous production of recombinant proteins are generally required to be easy to cultivate and should grow to high cell densities. Special needs concerning growth media or culture conditions narrow the benefit of a potential host organism as its handling might be expensive and labor intense. To test *P. stutzeri’s* applicability as a production host for membrane proteins, we first estimated the doubling time of the *P. stutzeri* cells at different temperatures between 20 and 40 °C, and compared its growth and viability in nutritionally rich (LB) and asparagine minimal (Asn) medium.

**Fig. 1:**
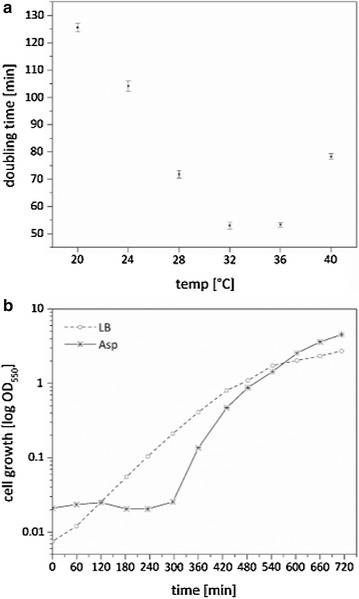
Growth of *Pseudomonas stutzeri*. **a** The doubling time of cultures grown in LB were compared at different temperatures. Each data point represents the mean value ± standard deviation (SD), which is calculated from three independent measurements. **b** Growth in nutritionally rich (LB) and asparagine minimal (Asn) medium at 32 °C

In LB medium, the *P. stutzeri* cell growth displayed a reverse bell-shaped dependence on temperature (Fig. 1a). The doubling time, which was determined from the half-logarithmic portion of the optical density in the exponential growth phase (OD_600_ = 0.2–0.8) was approximately 53 min at 32 °C. Increasing the temperature to 36 °C did not affect the cell proliferation rate, while decreasing the temperature from 32 to 28 °C prolonged the generation time to 72 min. Cell cultures reached OD_600_ values of 5–6 without obvious accumulation of dead cells after incubation overnight at 32 °C. Incubation at 40 °C, on the other hand, led to the formation of aggregates of dead cells in the stationary phase. Therefore, a growth temperature of 32 °C was used throughout the present work.

Besides the effect of different temperatures, the influence of the nutrient content on the growth rate was investigated by comparing the growth in LB and Asn medium (Fig. 1b). For this experiment, the cells were pre-cultured in LB medium prior to inoculation. In LB medium, the exponential growth phase occurred between 2 and 6 h after inoculation of the main culture (dotted line Fig. 1b). Before dividing, *P. stutzeri* cells needed 5 h to adapt to Asn medium (solid line Fig. 1b), which is a mineral base and contains asparagine as the source of nitrogen and carbon. For Asn medium, the doubling time was determined to be about 34 min, which is shorter than that observed in LB medium. However, due to the elongated lag phase, the stationary phase was reached simultaneously in both media after 10–11 h of incubation and the final OD values were approximately OD_600_ = 2 and 4 in LB and Asn medium, respectively. It should be noted that the prolonged lag phase can be prevented by using Asn medium for the pre-culture preparation.

The correlation between optical density at 600 nm and cell number was analyzed using a Neubauer chamber. Between OD_600_ of 0.3 and 0.6, it was found that one OD_600_ unit corresponds to 1.2 ± 0.3 × 10^9^ cells/ml.

### Sensitivity of *P. stutzeri* strain ZoBell to antibiotics

Antibiotic resistance genes are commonly used for selection in studies requiring the genetic manipulation of an organism, e.g. transformation, recombinant protein production or generation of gene deletion mutants. Previous studies have shown that isolates of *P. stutzeri* are sensitive to more antibiotics than the human pathogen *P. aeruginosa* that possesses a wide range of antibiotic resistance mechanisms [20]. To promote the usage of *P. stutzeri* as a heterologous protein production system, we systematically tested nine antibiotics that are routinely used in research laboratories for their activity against *P. stutzeri* ZoBell. Antimicrobial susceptibility tests were performed according to the European Committee on Antimicrobial Susceptibility Testing (EUCAST) gradient method. The minimal inhibitory concentration (MIC) of each antibiotic was assessed using MIC test strips and the activity was classified as sensitive or resistant according to the reference values listed for each antibiotic by EUCAST (18.12.2015).

Table 1 summarizes the determined MICs and the activity of the respective antibiotic agent. Narrow- and extended-spectrum cephalosporins and glycopeptides showed no activity against *P. stutzeri* in previous studies [20]. Therefore, cefazolin (CFZ) and vancomycin (VAN) were assumed to be inactive and were chosen as internal quality controls for the MIC test. As expected, the determined MICs of 32 µg/ml for CFZ and >256 µg/ml for VAN classified them as ineffective antimicrobial agents against *P. stutzeri* ZoBell. Ampicillin (AMP), chloramphenicol (CAM), gentamycin (GEN), kanamycin (KAN), streptomycin (STR) and tetracycline (TAC), on the other hand, were found to be active with MICs ranging from 0.19 to 6 µg/ml. The MIC of spectinomycin (SPC) was determined to be 48 µg/ml for *P. stutzeri* ZoBell and is higher than the value for the inactive CFZ (32 µg/ml). However, organisms are considered to be resistant to SPC at MICs above 64 µg/ml, therefore, SPC was classified as active.

**Table 1:** Minimal inhibitory concentration and minimal active concentration of all tested antibiotics

Class	Antibiotic	Disk diffusion		In solution
		Activity	MIC (µg/ml)	MAC (µg/ml)
β-Lactams	Ampicillin	S	1.0	>100
Cephalosporins	Cefazolin	R	32	nt
Miscellaneous	Chloramphenicol	S	2	50
Aminoglycoside	Gentamycin	S	0.38	5
Aminoglycoside	Kanamycin	S	1.0	10
Aminoglycoside	Spectinomycin	S	48	>100
Aminoglycoside	Streptomycin	S	6	25
Tetracyclines	Tetracycline	S	0.19	5
Glycopeptides	Vancomycin	R	>256	nt

R, resistant; S, sensitive; nt, not tested; MIC, minimal inhibitory concentration; MAC, minimal active concentration

All active compounds were also tested for their activity in liquid culture and the minimal active concentration (MAC) was determined (Table 1). GEN and TAC inhibited growth at the lowest tested concentration of 5 µg/ml. KAN and STR were active at 10 and 25 µg/ml, respectively. CAM affected growth at the lowest concentration of 5 µg/ml but full inhibition was only observed at 50 µg/ml. No activity was observed for AMP and SPC up to the highest tested concentration of 100 µg/ml. Therefore, according to our results, GEN, TAC, KAN, STR and CAM resistant genes could serve as selectable marker for the development of expression vectors or gene manipulation system. AMP and SPC are inactive and can be used as additive for the selection during the cultivation of *P. stutzeri* wild-type strain.

### DNA transformation into *P. stutzeri* by electroporation

For the expression of heterologous proteins, exogenous DNA containing the target gene has to be introduced into the host organism. Electroporation has been demonstrated to be an efficient method to deliver plasmid DNA into many Pseudomonas species [23]. In this study, we tested different *P. aeruginosa* electroporation protocols to prepare electrocompetent *P. stutzeri* cells [24–26]. Cells prepared in 300 mM sucrose could be transformed with efficiencies ranging from 10^3^ to 10^4^ cfu/μg DNA (Table 2). When *P. stutzeri* was cultivated in Asn instead of LB medium prior to preparation, a 10–100-fold decrease in transformation efficiency was observed. On the other hand, varying the DNA amounts, ranging from 50 to 500 ng, did not considerably affect the transformation efficiency.

**Table 2:** Electroporation of *P. stutzeri*

Donor strain	Culture medium for recipient strain (*P. stutzeri* ZoBell)	Treatment	Transformants per μg plasmid DNA
*P. stutzeri* ZoBell	LB medium	/	10^6^–10^7^
*E. coli* DH5a or JM110	LB medium	/	10^3^–10^4^
*E. coli* DH5a	Asn medium	/	10^2^–10^3^
*E. coli* DH5a	LB medium	In vitro methylation using *P. stutzeri* cell-free extracts^a^	10^3^–10^4^
*E. coli* DH5a	LB medium	Electroporated with TypeOne restriction inhibitor^b^	10^3^–10^4^

Electroporation was performed with 100 ng of plasmid DNA as described in “Electroporation of *Pseudomonas stutzeri*”

^a^Plasmid was treated with *P. stutzeri* cell-free extracts prepared as described [28, 29]

^b^TypeOne restriction inhibitor was purchased from Epicentre

We observed 100–1000-fold increased transformation frequencies with plasmid DNA prepared from *P. stutzeri* compared to those isolated from *E. coli* DH5α (dam^+^/dcm^+^). DpnI digestion of the plasmid DNA isolated from *P. stutzeri* revealed that DNA adenosine methylation (Dam methylation) is not present in *P. stutzeri*. This observation is in accordance with previous reports on other strains of *P. stutzeri* [27]. Because the transformation efficiency can be reduced when Dam-modified DNA is introduced into Dam^−^ species, non-methylated plasmid DNA was isolated from *E. coli* strain JM110 (dam^−^/dcm^−^) and used for the electroporation. However, no clear difference was observed between the plasmid DNAs isolated from the two *E. coli* strains.

To investigate whether the observed differences in transformation efficiency are attributable to the different methylation pattern, in vitro methylation was performed using cell-free extracts prepared from *P. stutzeri* cultures. Two published methods were applied [28, 29] but no improvement was observed. In addition, when plasmid DNA was also electroporated in the presence of TypeOne restriction inhibitor, again no increase in the transformation efficiency could be achieved.

### Construction of the broad-host-range vector pL2020

As pointed out in the previous sections, *P. stutzer*i can be cultivated under similar conditions as *E. coli*. Previously, plasmid-based gene expression was demonstrated, leading to high-yield production of recombinant *cbb*_3_-type cytochrome *c* oxidase in *P. stutzeri* [30]. In this case, expression was driven by the endogenous *cbb*_3_ oxidase promotor that is upregulated at low oxygen concentrations. However, this system does not allow a tight control of the expression level making it unfavorable for the recombinant overproduction of many membrane proteins.

**Fig. 2:**
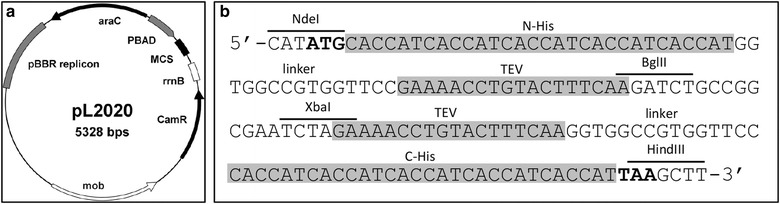
pL2020 is a broad host range vector enabling regulated and dose-dependent protein production in Gram-negative bacteria. It is based on the pBBR1MCS1 plasmid and utilizes the *araC*/P_BAD_ system. **a** Plasmid map of pL2020. *araC*, regulater protein; P_BAD_, l-arabinose inducible promoter; MCS, multiple cloning site; rrnB, transcription termination sequence; CamR, chloramphenicol resistance gene; mob, mobility element; pBBR, broad host range origin of replication. **b** Multiple cloning site of the pL2020 vector. The inverse architecture facilitates cloning of N- or C-terminally tagged His_10_ fusions. Upon purification the affinity tag can be removed by TEV cleavage

We therefore constructed the novel expression vector pL2020 capable of producing recombinant proteins in *P. stutzeri* (Fig. 2a). The 5328 base-pair vector is constructed based on the backbone of the broad-host-range plasmid pBBR1MCS1 [31]. The vector is stably maintained in many Gram-negative bacteria at low to medium copy numbers, including *E. coli* and *P. stutzeri* and is compatible with other broad-host-range vectors [32]. Instead of the endogenous oxidase promoter, pL2020 utilizes the *araC*/P_BAD_ system, which is known to be functional in many different Pseudomonas species [33] for inducible gene expression. In the absence of the inducer the transcription is repressed 1200-fold, while the expression of the gene of interest can be induced by l-arabinose at a wide range of concentrations [34]. In addition, resistance to chloramphenicol is conferred by a chloramphenicol acetyltransferase cassette, which allows efficient selection of positive transformants on agar plates. To facilitate the cloning and purification procedure, a new multiple cloning site (MCS) containing in-frame fusions of optional 5′ (N-terminal) and 3′ (C-terminal) His_10_-tag sequences, was designed and introduced downstream of the P_BAD_ promoter. Furthermore, two TEV protease sites were also included for the cleavage of the His_10_-tag after Ni–NTA affinity purification. For restriction enzyme-based cloning into pL2020, unique restriction sites NdeI, BglII, XbaI and HindIII were introduced (Fig. 2b). The pL2020 vector was submitted to Addgene (http://www.addgene.org; Deposit Number: 73706).

### Recombinant production of membrane proteins in *P. stutzeri*

To test the applicability of *P. stutzeri* for the production of recombinant membrane proteins, a total of 44 membrane proteins were chosen as a test set (Table 3). Thirty-six of these proteins are secondary active transporters selected from 14 different protein families and three source organisms. These proteins were used as a reference to evaluate the performance of *P. stutzeri* as an expression host and to compare the expression level with previous results obtained with *E. coli* [35]. Additionally, we have chosen 8 membrane transporters from the human pathogen *P. aeruginosa*. These proteins are assigned to resistance and/or biofilm formation according to the Pseudomonas Genome Database [36]. Due to the close systematic relationship, *P. stutzeri* can be considered as a “quasi homologous” production system for the production of the *P. aeruginosa* proteins.

**Table 3:** Production levels in *P. stutzeri* of all 44 tested membrane transporters

Source organism	Locus tag	Family	GI number	Production level	
				C-His	N-His
*P. aeruginosa* ^a^	PA1247	ABC	9947178	□	++
*A. aeolicus* ^b^	Aq_1392	AEC	15606582	□	NT
*P. furiosus* ^c^	PF0449	AEC	33359482	□	NT
*S. enterica* ^d^	STM006	AGCS	16418499	□	NT
*P. furiosus*	PF0514	AGCS	18976886	□	NT
*S. enterica*	STM0700	APC	16419208	++	NT
*S. enterica*	STM0969	APC	16419480	+	++
*S. enterica*	STM1477	APC	16419996	+	+
*S. enterica*	STM2200	APC	16420738	++	NT
*S. enterica*	STM2357	APC	16420900	+	+ +
*S. enterica*	STM3225	DAACS	16421782	++	NT
*A. aeolicus*	Aq_1330	DAACS	15606533	++	NT
*A. aeolicus*	Aq_031	DASS	15605634	□	+
*S. enterica*	STM3166	DASS	16421721	□	NT
*S. enterica*	STM3356	DASS	16421915	□	NT
*S. enterica*	STM0832	DMT	16419338	+	++
*S. enterica*	STM3765	DMT	16422334	+	+ +
*S. enterica*	STM3746	ESS	16767031	+	+ +
*S. enterica*	STM2913	GntP	16421462	++	NT
*S. enterica*	STM3512	GntP	16422071	+	++
*S. enterica*	STM3541	GntP	16422100	+	+
*S. enterica*	STM3801	GntP	16422374	+	++
*S. enterica*	STM4482	GntP	16423047	□	NT
*P. aeruginosa*	PA3553	GTT	9949705	+ +	NT
*P. furiosus*	PF0520	MFS	18976892	□	+
*S. enterica*	STM1360	MFS	16764711	++	NT
*P. aeruginosa*	PA1236	MFS	9947166	□	+ +
*P. aeruginosa*	PA1569	MFS	9947531	++	NT
*A. aeolicus*	Aq_851	MIT	15606202	++	NT
*P. furiosus*	PF2036	MIT	18978408	++	NT
*P. aeruginosa*	PA2241	MOP	9948266	++	NT
*S. enterica*	STM0522	NCS1	16419032	□	□
*S. enterica*	STM3333	NCS1	16421891	++	NT
*P. furiosus*	PF0852	NCS2	18977224	□	NT
*P. furiosus*	PF1240	NCS2	18977612	++	NT
*S. enterica*	STM0524	NCS2	16419034	+	□
*S. enterica*	STM2497	NCS2	16421039	++	NT
*S. enterica*	STM3631	NCS2	16422196	++	NT
*A. aeolicus*	Aq_2077	NSS	15607041	□	NT
*P. aeruginosa*	PA2760	Opr	15597956	++	NT
*P. aeruginosa*	PA1436	RND	9947386	++	NT
*P. aeruginosa*	PA2495	RND	9948547	++	NT
*A. aeolicus*	Aq_1504	Trk	15606659	+	□
*S. enterica*	STM3986	Trk	16422552	++	NT

++, ≥0.1 µg/ml; +, <0.1 µg/ml; □, no protein detected; NT, not tested; AEC, Auxin Efflux Carrier family; AGCS, Alanine/Glycine:Cation Symporter family; APC, Amino Acid-Polyamid-Organocation family; DAACS, Dicarboxylate/Amino Acid:Cation Symporter family; DASS, Divalent Anion:Na^+^ Symporter family; DMT, Drug/Metabolite Transporter superfamily; ESS, Glutamate:Na^+^ Symporter family; GntP, Gluconate:H^+^ Symporter family; GTT:Glycosyl Transferase Transporter superfamily; MFS, Major Facilitator Superfamily; MIT, CorA Metal Ion Transporter family; MOP, Multidrug/Oligosaccharidyl-lipd/Polysaccharide Flippase superfamily; NCS1: Nucleobase:Cation Symporter-1 family; NCS2, Nucleobase:Cation Symporter-2 family; NSS, Neurotransmitter:Na^+^ Symporter family; Opr, Outer Membrane Porin family; RND, Resistance-Nodulation-Cell Devision superfamily; Trk, K^+^ Transporter family

^a^ *Pseudomonas aeruginosa* PAO1

^b^ *Aquifex aeolicus* VF5

^c^ *Pyrococcus furiosus* DSM 3638

^d^ *Salmonella enterica* subsp. enterica serovar Typhimurium str. LT2

In a first attempt, all proteins were cloned with a C-terminal His_10_-tag. Cells were collected 2 and 4 h after induction with four different concentrations of l-arabinose between 0.2–0.0002% (w/v) and tested for the production of the target proteins by immunodetection with an anti polyhistidine antibody using the dot blot method (Additional file 1: Figure S2). The production levels were compared to a standard of 50 ng of His-tagged GFP spotted on the same membrane. Signals with lower intensity were classified as low-production, signals with comparable or higher intensity as high-production corresponding to a calculated yield of at least 0.1 mg of recombinantly produced protein per liter of culture medium.

Of the C-terminally tagged heterologous proteins 11 out of 36 (11/36) were produced at low levels and 13 (13/36) were produced at high levels. As the position of the tag may influence the production of proteins [35], 14 (14/36) transporters not being produced at high levels in the initial screen were further cloned with an N-instead of a C-terminal His_10_-tag. This change of the tag position increased the production of seven proteins from low- to high-level production. Two further proteins not produced with a C-terminal tag before could be produced at low levels. Only for five proteins no improvement or even a decrease of the production level was observed. Taken together, 6 (6/36 = 16%) heterologous proteins were produced at low levels and 20 (20/36 = 56%) were produced at high levels corresponding to an overall success rate of 72%.

We used the same screening procedure for the additional eight proteins selected from *P. aeruginosa* and considered their production as a “quasi homologous” system. All proteins were produced at high levels, 6 (6/8) in the first attempt with a C-terminal tag, the remaining 2 (2/8) with the N-terminal version.

In summary, out of the 44 (36 heterologous + 8 “homologous”) proteins 34 (34/44 = 77%) were detected in the dot blot. Of these 34 produced proteins, 28 (28/44 = 63%) were produced at high levels. Only for 10 (10/44) proteins, no production was detectable in *P. stutzeri*, all of them being heterologous targets from different families and source organisms.

Our serial cloning strategy yielded 44 constructs with a C-terminal His_10_ tag and additional 16 with the N-terminal variants. These 60 constructs were analyzed for protein production under 8 conditions (2 time points and each with 4 inducer concentrations) giving a total of 480 samples collected (Additional file 2: Figure S3). From these data none of the eight conditions stands out with respect to success rate. However, there is a tendency that more proteins were produced at high inducer concentrations of 0.2 or 0.02% l-arabinose. Differences between the two time points (2 and 4 h after induction) are even less pronounced (Additional file 3: Table S3). For some proteins high-level production was only detected 4 h after induction. On the other hand, for none of the proteins high-level production was detected solely 2 h after induction. Therefore, in an initial production test the screening of different inducer concentrations in parallel at a single time point 4 h after induction is sufficient to identify potential target proteins for further studies.

### GFP folding reporter assay

**Fig. 3:**
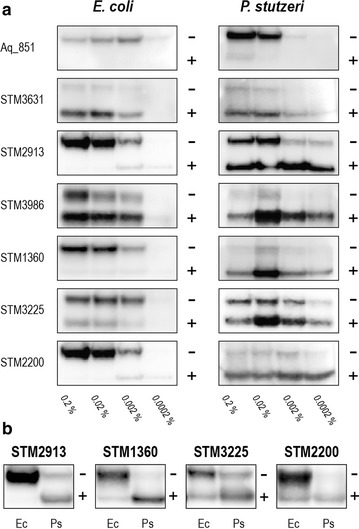
GFP folding assay in *E. coli* TOP10 and *P. stutzeri* ZoBell. **a** Production of seven GFP-fused membrane proteins was induced with four different l-arabinose concentrations (w/v). 35 µg of total protein were analyzed on SDS-PAGE, followed by in-gel fluorescence detection and Western blotting performed against the His tag. The “+” and “−” signs indicate the positions of fluorescent and nonfluorescent species of the proteins, respectively. **b** Direct quantitative comparison of protein folding in *E. coli* (Ec) and *P. stutzeri* (Ps). Protein production was induced with 0.02% (w/v) l-arabinose

In this study, the green fluorescent protein (GFP) was used as a folding indicator to distinguish between the well-folded and unfolded protein species. For the GFP folding assay, seven heterologous proteins that could be produced at relatively high levels with a C-terminal His_10_-tag in *P. stutzeri* were selected, and the DNA encoding the GFP moiety was inserted between the protein coding sequence and the His_10_-tag (Additional file 2: Figure S3). For comparison, the GFP fusion constructs were introduced into *P. stutzeri* and *E. coli* TOP10 cells, respectively. The production of the target proteins was induced by l-arabinose in various concentrations (0.2, 0.02, 0.002, and 0.0002%), and the amount of well-folded or aggregated protein was estimated by the detection of in gel fluorescence and immunodetection with antibodies against the His_10_-tag (Fig. 3).

For all proteins produced in *P. stutzeri*, Western blotting revealed the presence of two immunopositive bands. The strong GFP fluorescence signal could be observed only for the lower band representing the properly folded protein species, whereas the upper nonfluorescent band consisted of unfolded protein [37]. For two transporter proteins (Aq_851 and STM3631) produced in both expression hosts, no substantial difference was detected between *P. stutzeri* and *E. coli*. Aq_851 did not produce fluorescence signals but was detectable as unfolded species on the Western blot. On the other hand, for STM3631 a fluorescent band was detectable illustrating that the protein was mainly folded in both hosts. For the remaining five proteins (STM2913, STM3986, STM1360, STM3225 and STM2200), both, the properly folded and aggregated protein species were observed, and the ratios between them varied greatly from *E. coli* to *P. stutzeri*. In *E. coli*, with the exception of STM3986, the majority of the proteins were present as unfolded and aggregated species. In contrast, *P. stutzeri* was able to produce all five proteins in a mostly folded state with at least one inducer concentration tested (Fig. 3a).

To avoid variation of immunostaining intensity of individual blots, a direct comparison of the folding pattern of four proteins was performed (Fig. 3b). Samples from *E. coli* and *P. stutzeri* induced with 0.02% (w/v) l-arabinose were loaded onto the same gel and analyzed further, because this inducer concentration gave the most pronounced effects on protein folding. For three of the proteins (STM2913, STM1360 and STM3225), the proportion of the fluorescent and properly folded protein was clearly increased when *P. stutzeri* was used as the production host. For STM2200, no substantial difference regarding the amount of the folded protein was observed between *E. coli* and *P. stutzeri*, however, a large fraction of the nonfluorescent and unfolded species was present in *E. coli* (Fig. 3b).

### Large-scale production and purification of STM2913

**Fig. 4:**
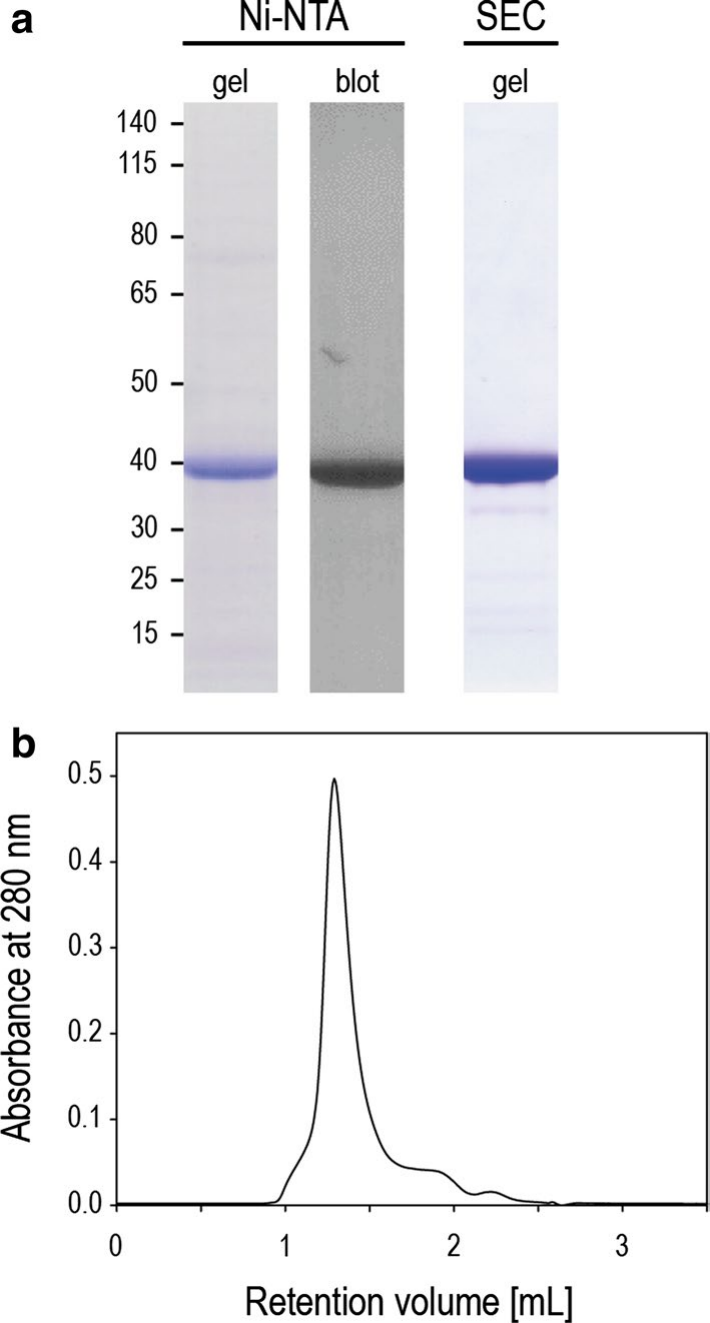
Large-scale purification of STM2913 produced in *P. stutzeri*. STM2913 from *S. enterica* was heterologously produced in *P. stutzeri* and purified by Ni–NTA affinity chromatography followed by size exclusion chromatography (SEC). **a** SDS-PAGE analysis of purified STM2913. Purity was analyzed by Coomassie staining and identity of STM2913 was confirmed by poly-histidine immunodetection. Gel, Coomassie stained SDS-PAGE; Blot, Western blot with poly-histidine antibody. Molecular weights are indicated in kDa on the left of the gel. **b** Gel filtration profile for STM2913 on a Superdex 200 column

The GntP (Gluconate:H^+^ Symporter) family transporter STM2913 derived from *S. enterica* was chosen for the large-scale production in *P. stutzeri*. In a previous study using *E. coli* as the production host, this protein could not be produced at high levels [35]. Production of the recombinant STM2913, with a C-terminal His_10_-tag, was induced with 0.02% l-arabinose. Membranes prepared from 6 l of *P. stutzeri* culture were solubilized with *n*-dodecyl β-d-maltoside (β-DDM), and heterologously produced STM2913 was purified on a Ni–NTA (nickel–nitrilotriacetic acid) affinity column followed by Superdex 200 size exclusion chromatography (SEC). The eluted protein was analyzed by SDS-PAGE and its identity was confirmed by immunoblotting using an anti polyhistidine antibody (Fig. 4a). Upon Coomassie staining, a single prominent band was observed at a position corresponding to a molecular weight of 40 kDa, which is smaller than the molecular mass of 58.6 kDa deduced from the STM2913 coding sequence including the His_10_-tag. The “gel shifting” is frequently observed for membrane proteins [38] and is explained by an increased binding of SDS to the proteins [39]. Despite the anomalous migration behavior, SDS-PAGE analysis revealed the high purity of the isolated STM2913. In addition, a symmetrical elution peak in the gel filtration chromatogram indicates a homogeneous protein-detergent complex preparation. The final yield of the purified proteins was approx. 0.2 mg/l of culture. This yield is sufficient for most biochemical analyses as well as for crystallization studies.

## Discussion

### Microbiological features of bacterial production hosts

Membrane proteins are important drug targets and play key roles in many different cellular processes, bringing them into the focus of many research projects investigating their structure and function. The production and purification of sufficient quantities of the protein of interest in a properly folded state is a crucial prerequisite for structural and functional studies underlining the importance of a suitable production system. Due to its easy handling, relatively low cost and the accessibility of a variety of expression vectors and strains *E. coli* is by far the most commonly used prokaryotic host for recombinant membrane protein production. Great efforts have been made to improve the success with *E. coli* systems, however, production of many membrane proteins in sufficient yields still frequently fail. Therefore, alternative prokaryotic hosts have been tested for their applicability for membrane protein production but only very few have been found to be successful in a similar extent as *E. coli*. Among them, the most widely spread are the Gram-positive bacteria *L. lactis* and *B. subtilis* [40, 41]. Both organisms are comparable to *E. coli* with respect to growth rate and cultivation conditions but differ in membrane architecture and at least in parts in the composition of their membrane protein folding and insertion machinery [2, 14].

Previously, Surade et al. compared the production of 37 secondary active transporters in *E. coli* and *L. lactis* and found the latter to be a less suitable host for the production of this set of proteins [35]. It was mentioned elsewhere [15] that the better performance of *E. coli* might be explained by the selection of proteins mostly derived from Gram-negative sources. Following this line of argument Gram-positive hosts cannot be considered as convincing alternatives to *E. coli*, at least for the production of proteins derived from Gram-negative sources.

A few Gram-negative organisms have been examined for the production of recombinant proteins. In particular, a *P. fluorescens* based expression system has been developed [42] and commercialized (http://www.pfenex.com). *Pseudomonas fluorescens* can be cultivated to high cell densities in bioreactors and this expression platform has been extensively employed for the high-yield production of soluble and/or secreted pharmaceutical proteins. However, *P. fluorescens* has not been systematically tested as a host for integral membrane protein expression and therefore its capability remains limited.

In this study, we used *P. stutzeri* ZoBell, a nonfluorescent Pseudomonas species, as an alternative platform for the production of recombinant membrane proteins. This bacterium has been routinely used in our laboratory for years to study the structure and function of its membrane-embedded respiratory enzymes. Several features are notable concerning its microbiological properties: (i) *P. stutzeri* ZoBell has a medium-sized genome of 4.9 Mb and the draft genome sequences are available [43]. (ii) The species has fast growth kinetics with a doubling time of approximately 35 min under optimal growth conditions. This doubling time is slightly longer than that of *E. coli* but still fast enough to grow cultures within 1 day. It should be noted that a slower growth rate might have a beneficial effect on the production of membrane protein because it allows to properly fold newly transcribed recombinant proteins and to correctly insert “difficult folders” into the membrane [38]. (iii) *P. stutzeri* can be cultivated on simple and inexpensive media reaching high cell densities (OD_600_ > 5). It is capable of utilizing a variety of compounds as sole carbon and nitrogen source [20]. A chemically defined medium, e.g. Asn minimal medium, would provide the possibility for studies requiring the incorporation of isotopic labels. (iv) The bacterium is well known for its ability to switch from aerobic respiration to denitrification to gain energy. Thus, *P. stutzeri* system can be potentially used for the expression of oxygen-sensitive proteins under anaerobic conditions. (v) Directed disruption of chromosomal genes can be done in *P. stutzeri* using a suicide plasmid-based method [30] or the lambda Red recombinase system [44]. (vi) Electroporation can be used for the introduction of expression vectors into the cells. Although the transformation frequency was relatively low if the plasmid DNA was isolated from *E. coli* the efficiency is still sufficient to reliably obtain positive transformants. As reported before the uptake of DNA is often hindered by a restriction/methylation barrier between different species [28, 29, 45]. Several such methylation/restriction systems are predicted to be present in *P. stutzeri* by the REBASE database [46]. The in vitro protection of the DNA prior to transformation or the generation of restriction-deficient strains might be a suitable strategy to increase the transformation efficiency in future studies.

### A single plasmid based work flow for membrane protein production

**Fig. 5:**
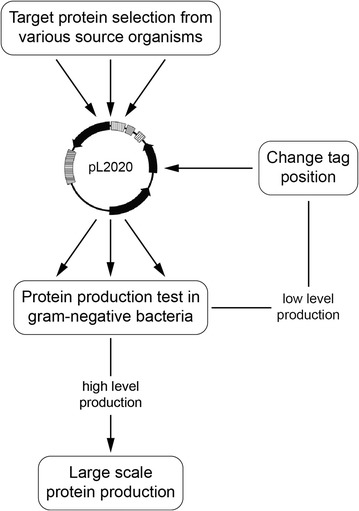
Workflow to screen membrane protein production. The screening procedure is based on a single vector capable to drive expression in several Gram-negative hosts. Due to the serial cloning strategy the number of generated constructs is reduced to a minimum. The workflow was applied with *P. stutzeri* as the production host and yielded 20 out of 36 proteins to be produced at high levels

To overcome the challenge of low success rates for membrane protein production, fast and efficient screening protocols are crucial to identify suitable target proteins for subsequent studies. Many approaches have been suggested to test different promoter systems, protein fusions or hosts in parallel. These protocols usually require intensive cloning work and the number of generated constructs rapidly exceeds the number of tested proteins multiple times [35, 47, 48]. For this study, we created a workflow following a serial cloning strategy minimizing the number of constructs that have to be generated (Fig. 5). All proteins were initially cloned with a C-terminal His_10_-tag. The C-terminal localization of the tag is considered to be favorable as it does not interfere with the membrane insertion of the N-terminus and was previously reported to perform better than N-terminal fusions [35]. In addition, we selected 16 proteins that were not produced at high levels with a C-terminal His_10_-tag and changed the tag position to the N-terminus (Additional file 3: Table S3). Using this approach the number of proteins produced at high levels increased from 19 to 28 (28/44). As only a subset of proteins was tested with both tags, our data do not allow a general conclusion about the more advantageous tag position, but clearly the overall success rate for protein production was improved. This serial cloning strategy reduces the cloning work to a minimum and was facilitated by the MCS of the pL2020 vector (Fig. 2b). Its inverse architecture easily allows to create both, N- and C-terminally tagged variants of the target protein simplifying the cloning procedure.

We applied the strategy to the new production host *P. stutzeri*, but functionality of the pL2020 vector could also be demonstrated for *E. coli* (Fig. 3). As the pBBR origin of replication and the P_BAD_ promoter were found to be functional in other Gram-negative species [31, 33] the number of host organisms could be further extended in future studies, e.g. proteins from pathogenic organisms can be produced in nonpathogenic species of the same genus. In our study, some proteins were found to be produced at high levels in *P. stutzeri* but not in *E. coli* and vice versa. This increase of the overall success rate demonstrates the benefit of using different production hosts in parallel. If a single broad-host-range plasmid as the pL2020 vector is used, no additional cloning work is required.

Besides the broad-host-range, the pL2020 vector also possesses the advantage of a tightly controlled gene expression from the P_BAD_ promoter. Toxicity of membrane proteins is at least in some cases believed to be caused by an overwhelming of the membrane insertion machinery or a disruption of the energy generation by “flooding” the membrane with extrinsic proteins [49]. Mutated *E. coli* strains selected for their ability to produce toxic proteins were found to carry mutations lowering the protein production level [10]. These findings illustrate the importance of a tightly regulated gene expression as it is facilitated by the P_BAD_ promoter [34]. In *E. coli*, the *araC*-P_BAD_ system has been shown to suffer from “all-or-none” gene expression at intermediate induction levels, due to the presence of the arabinose transporter AraE [50–52]. However, it has been reported that *P. stutzeri* is unable to metabolize l-arabinose [53] and homologs of the araBAD and araE genes are not present in its genome according to BLAST analysis. Although, in this study, we did not investigate whether gene expression from P_BAD_ promoter is titratable with respect to individual cells, dose-dependent expression response was clearly observed within the concentrations tested (0.0002–0.2% l-arabinose) (Fig. 3; Additional file 1: Figure S2). Considering all 44 tested proteins, inducer concentrations of 0.2 or 0.02% (w/v) l-arabinose were found to lead to high production levels for most target proteins (Additional file 4: Figure S1). Nevertheless, in some cases no production was detectable at these concentrations whereas lower concentrations led to production or even high-level production. Screening different inducer concentrations, therefore, was an efficient strategy to increase the number of produced target proteins.

On the other hand, comparison of different time points after induction had a negligible influence on the production levels (Additional file 3: Table S3) and we suggest to exclude it from the initial screening procedure to further speed up the screening process in future studies.

### Recombinant production of membrane proteins

We tested the production of 36 heterologous secondary active membrane transporters in the new production host *P. stutzeri*. We compared the results to a study of Surade et al. (Additional file 5: Table S2) who used the same set of proteins to investigate their production in *E. coli* and *L. lactis* [35]. The proteins were selected from 14 families of secondary active transporters and the three source organisms *S. enterica*, *A. aeolicus* and *P. furiosus*.

In our study, we found a comparable success rate as for *E. coli*. In fact, the number of proteins produced at high levels increased from 16 in *E. coli* to 20 in *P. stutzeri*. Only 13 proteins were produced in both hosts at high levels. Seven of the 36 heterologous proteins were produced at high levels in *P. stutzeri* but not in *E. coli*.

Whereas production of the same test set in *L. lactis* did not improve the overall success rate, the use of *P. stutzeri* as an alternative production host did increase the number of target proteins produced at high levels from 16 to 23. Moreover, only four proteins could not be detected in *E. coli* nor in *P. stutzeri* resulting in an overall success rate of 32 (32/36) proteins (89%) being produced at low or high levels in *E. coli* and/or *P. stutzeri*.

It should be noted that certain protein families appear to be produced more likely in either *E. coli* or *P. stutzeri*, e.g. the Gluconate:H^+^ Symporter (GntP) family. Five members of the family derived from *S. enterica* were included in the study and could be produced only at low levels in *E. coli*. In contrast, *P. stutzeri* yielded three of the proteins at high levels. In particular in structural studies the focus of the project is often on a protein family rather than on a certain protein. In cases in which a protein family repeatedly fails to be produced in *E. coli* the new host *P. stutzeri* is an attractive alternative. Overall, from 12 of the 14 protein families at least one member could be produced at high levels in *E. coli* and/or *P. stutzeri*.

*Pseudomonas stutzeri* has proven to be a suitable host for the heterologous production of membrane proteins from different sources. However, proteins from the most closely related source *S. enterica* performed better than those derived from the Gram-positive *A. aeolicus* or the archaeon *P. furiosus*. We therefore used our new production system for proteins derived from the human pathogen *P. aeruginosa*, a close relative of *P. stutzeri* [22]. The genus Pseudomonas comprises many species involved in biologically and clinically relevant processes and a production system with high success rates in membrane protein production for these species would be of great advantage. Eight membrane proteins selected from *P. aeruginosa* were tested, considered as “quasi homologous” production. Indeed, with *P. stutzeri* all “quasi homologous” targets from *P. aeruginosa* could be obtained at high levels.

Madhavan et al. have tested the production of 87 *P. aeruginosa* membrane proteins in *E. coli* and reported production of 61 of the proteins [38]. However, only 25 proteins were scored with “high expression” and reduction of the growth temperature to slow down the translation rate was required for some of the proteins to enable their production and prevent an overloading of the translocon. *P. stutzeri* on the other hand was grown at its optimal growth temperature and protein production was achieved with the highest tested inducer concentrations leading to strong gene expression and hence high demands on the translocation machinery. More comprehensive experiments are necessary but our results suggest that *P. stutzeri’s* membrane protein folding and insertion machinery, not surprisingly, is more adapted to *P. aeruginosa* proteins than that of *E. coli*. Even though both organisms belong to the class of gammaproteobacteria it appears the physiological differences are sufficient to hamper protein production in *E. coli* even for proteins derived from closely related sources.

### Protein purification and analysis

Despite the high success rate, recombinant production of membrane proteins often results in the formation of aggregates of unfolded or misfolded proteins. Previously, a method based on the detection of the GFP fluorescence signal has been developed to distinguish between folded and unfolded GFP fusion proteins [37, 54]. In this work, we adopted this strategy, using C-terminal GFP fusions, to monitor the folding process of recombinantly produced membrane proteins in *P. stutzeri*. Our results indicate that, indeed, misfolded or aggregated proteins can be detected, and they are probably expressed in the form of inclusion bodies. Nevertheless, compared to *E. coli*, a higher portion of well-folded protein can be obtained for several of the secondary active transporters in *P. stutzeri* (Fig. 3). Although it must be admitted that differences in the folding rates observed between *E. coli* and *P. stutzeri* may not be statistically significant, our results still imply that *P. stutzeri* is suitable for the heterologous production of membrane proteins.

In addition, the GntP family transporter STM2913 from *S. enterica* was chosen for the large-scale production and we could purify at least 1 mg of the protein from isolated membranes from 6 l of bacterial culture. Considerable amounts of the proteins were located in the membrane and could be solubilized with the relatively mild detergent DDM. As DDM does not efficiently solubilize misfolded proteins [37, 55], a proper membrane insertion of STM2913 was demonstrated. Even though high yields in the milligram range are favorable for functional and structural studies, many biophysical and biochemical techniques nowadays require comparably small quantities of purified protein. DLS and DSC measurements can be performed with sample volumes of 10 µl or less [56, 57] and the required sample amounts for certain ITC devices have been substantially downscaled. Automated crystallization facilities allow to set up 96-well screens with <100 µg of protein at concentrations of 10 µg/µl [58]. These techniques allow the biophysical and structural characterization of even small amounts of purified protein. Therefore, also targets produced at moderate amounts might be considered for functional and structural studies. Only 4 (4/36) of the tested heterologous target proteins could not be produced in any of the two tested hosts, further underlining the usefulness of *P. stutzeri* as a new alternative production host. Potentially, 32 out of the 36 tested proteins could be purified and therefore become accessible for future studies.

### Conclusions

As the production of sufficient amounts of protein still represents a major bottleneck in membrane protein research, new strategies to overcome this hurdle are of high interest to the field. Great efforts have been made to adopt existing production hosts to the challenge of membrane protein production. Certainly, with the increasing understanding of the biogenesis of membrane proteins more advanced and specialized strains can be and have been designed. It still appears that the applied strategies are not suitable for all types of proteins and some may possess intrinsic properties which make them difficult to produce in the existing systems.

In our study, we investigated *P. stutzeri’s* applicability as a host for the recombinant production of secondary active membrane transporters and compared its performance with the most common production host *E. coli*. Both organisms have a similar physiology with respect to growth rate and nutrient requirements. Therefore, *P. stutzeri* can be easily established in every research laboratory as a new alternative production system. Success rates for protein production are comparable to *E. coli* but can be increased if both hosts are used in parallel (Additional file 5: Table S2).

Based on the inducible broad-host-range vector pL2020 constructed for this work, an efficient work flow can be applied to screen protein production in *E. coli*, *P. stutzeri* and other potential (Gram-negative) production hosts in parallel (Fig. 5). No specialized expression constructs have to be created for each host separately. Thereby, time consuming cloning work is reduced.

We consider *P. stutzeri* as a true alternative to *E. coli* suitable to make proteins accessible for functional and structural research not being produced in sufficient amounts in the existing systems before.

## Methods

### Materials

*Pseudomonas stutzeri* strain ZoBell was used throughout the present study. *Escherichia coli* DH5α was used for general cloning purposes. *Escherichia coli* JM110 was used to propagate non-methylated DNA. Genomic DNA from *P. aeruginosa*, *Salmonella typhimurium* and *Pyrococcus furiosus* were purchased from Leibniz Institut DSMZ-Deutsche Sammlung von Mikroorganismen und Zellkulturen GmbH (http://www.dsmz.de). Genomic DNA from *Aquifex aeolicus* was isolated using the Epicentre QuickExtract DNA extraction solution. Synthetic oligonucleotides, obtained from Eurofins Genomics, are listed in Additional file 6: Table S1. *Pseudomonas stutzeri* cells were grown in lysogeny broth (LB) medium or in asparagine (Asn) minimal medium [30]. OD_600_ values were measured with an “Ultrospec 2100 pro” photometer (Amersham Biosciences).

### Determination of optimal growth temperature

To determine the optimal growth temperatures for *P. stutzeri*, a single colony (1–1.5 mm diameter) from a streaked LB agar plate containing 100 µg/ml ampicillin was used to inoculate antibiotic-free LB medium and cultured at 32 °C and 160 rpm for 18 h. This pre-culture was used to further inoculate 50 ml of LB medium in 100-ml unbaffled culture flasks to an OD_600_ of 0.1. Cultures were grown at different temperatures (20, 24, 28, 32, 36 and 40 °C) with shaking at 160 rpm. The growth was monitored every 30 min by measuring the optical density at 600 nm of the culture. For each temperature condition, three independent measurements were performed.

### Determination of cell number

The total cell numbers were counted using a Neubauer chamber and the phase contrast microscope. Cells collected at different OD_600_ values were diluted to ensure that each square of the Neubauer chamber contained 20–50 cells and the numbers were averaged by a count of four random squares.

### Antibiotic susceptibility test

The minimum inhibiting concentration (MIC) tests were performed following the EUCAST (European Committee on Antimicrobial Susceptibility Testing) guidelines. *Pseudomonas stutzeri* cells were picked up from an over-night LB agar plate and resuspended in sterile saline solution (100 mM NaCl) to an optical density of 0.5 McFarland standard. The inoculums were streaked onto Miller-Hinton agar plates (Bestbion) using sterile cotton swabs and left for drying for 10 min, followed by the application of MIC test strips (Bestbion). After 20 h of incubation at 35 °C, the plates were read visually and the MIC was recorded as the lowest concentration that inhibited the visible growth. The *E. coli* strain ATCC 25922 was used as a quality control reference strain for the susceptibility test.

In addition to the gradient-diffusion method, antibiotic resistance profiles were also determined by measuring the ability of *P. stutzeri* ZoBell cells to grow in the LB medium in the presence of each of several antibiotics at different concentrations. The growth was monitored by measuring the optical density at 600 nm.

### Construction of the expression vectors

To construct an inducible expression vector for *P. stutzeri*, the cloning was performed as follows: (1) a 1608-bp DNA fragment containing the *araC*-P_BAD_ repressor-promoter assemblage, a multiple cloning site (MCS) and the rrnB transcriptional terminator, was amplified by PCR using the pBAD-A2 vector [35] as template with primer pair #3/4 (Additional file 6: Table S1); (2) a second 3698-bp DNA fragment including the pBBR replicon, the mobilization (mob) gene and chloramphenicol resistance (Cam^R^) gene, was PCR amplified using the pBBR1MCS-1 vector [31] as template with primer pair #1/2; (3) the two DNA fragments were joined using the InFusion ligation-independent cloning method (Clontech), resulting in pL2010; (4) the vector pL2010 was modified by replacing the MCS. The new MCS-2 with a 15-bp overhang at both ends homologous to the vector sequence flanking the old MCS was synthesized by Eurofins Genomics. Primer pair #7/8 was used to amplify MCS-2 and to fuse it to the vector by InFusion cloning. The vector for this reaction was amplified with primer pair #5/6.

For the heterologous production of membrane transport proteins in *P. stutzeri*, the transporter genes were amplified either from genomic DNA or constructs described elsewhere [35], using Phusion DNA polymerase with primer pairs listed in Additional file 6: Table S1. The target genes were cloned into the pL2020 vector using the InFusion ligation-independent cloning method (Clontech). The final constructs were verified by DNA sequencing and introduced into *P. stutzeri* by electroporation.

### Electroporation of *Pseudomonas stutzeri*

Electrocompetent cells of *P. stutzeri* were prepared according to the slightly modified procedure of Choi et al. [24]. Briefly, 1-ml of cells in the early stationary phase (OD_600_ = 1.5–2.0) from cultures grown in LB medium were harvested by centrifugation at 5000×*g* and washed twice with 1 ml of room temperature (RT) 300 mM sucrose. Cells resuspended in 100 µl 300 mM sucrose were mixed with 200 ng plasmid DNA in a 1 mm electroporation cuvette. High voltage electroporation was performed using a Bio-Rad Gene Pulser at 25 µF, 200 Ω and 2.5 kV. After applying the pulse, 1 ml of SOC medium was added immediately and the cells were transferred to a culture tube and incubated at 37 °C for 1 h. Cells were plated on LB agar plates with appropriate antibiotic and incubated at 32 °C for 48–72 h.

### Small-scale protein production and dot blot assay

Small-scale expression trials were conducted using wild-type *P. stutzeri* transformed with the pL2020 vector carrying the gene of interest. A 100-ml culture derived from a single transformant was grown at 32 °C in Asn medium containing 34 µg/ml chloramphenicol (CAM) overnight. The overnight culture was used to inoculate 100 ml of Asn media containing 34 µg/ml CAM to an initial OD_600_ of 0.1. Cells were cultured at 32 °C and 160 rpm until the OD_600_ reached 0.5–0.6. Cultures were divided into five equal parts (15-ml culture in 100 ml baffled flask). One served as untreated control, while production of the target proteins in the other four cultures was induced by the addition of 0.2, 0.02, 0.002 and 0.0002% (w/v) l-arabinose (final concentration), respectively. The l-arabinose stock solutions were prepared freshly prior to use. Two and four hours after induction, 1 ml of each culture was harvested by centrifugation at 4 °C and 5000×*g* for 3 min and cell pellets were immediately frozen at −20 °C until use.

For protein expression analysis, the cell pellet was resuspended in 200 µl lysis buffer (50 mM Tris pH 8.0, 1 mM MgCl_2_, 0.1 mg/ml 4-(2-aminoethyl) benzene sulfonyl fluoride hydrochloride [Pefabloc], 2.5 U/ml Benzonase and 0.2 mg/ml lysozyme) and incubated at RT for 20 min. 600 µl 8 M urea were added followed by further incubation at RT for 20 min. The samples were spun down at 20,000 × g and 4 °C for 10 min. For dot blot assays, 300 µl of the supernatants were loaded on a PVDF membrane, blocked with 5% (w/v) milk powder in PBS buffer for 2 h at RT. Immunodetection of the His_10_-tagged protein was conducted using a monoclonal α-poly-histidine alkaline phosphatase conjugated antibody (Sigma) following the manufacturer’s instructions. The NBT-BCIP system was used for detection of the alkaline phosphatase. To evaluate the signal intensity, 0, 25, 50, 100, 200, 400, 600 and 800 ng purified His-tagged enhanced green fluorescent protein (EGFP) was used as a standard.

### GFP folding assay

The GFP folding assay was performed as described previously [37]. To construct the C-terminally GFP fused expression vectors, the GFP encoding DNA was inserted between the TEV protease cleavage site and His_10_ tag via InFusion cloning (Additional file 2: Figure S3). The resulting vectors were introduced into *P. stutzeri* by electroporation. For comparison, the same vectors were also transformed into *E. coli* strain TOP10. Cells were cultivated as described for the small-scale protein production with the exception that LB medium was used for *E. coli* cells. Cell pellets corresponding to 1 mg of total protein were collected 4 h after induction. Pellets were resuspended in 400 µl of ice-cold buffer [50 mM HEPES pH 8.0, 10% (v/v) glycerol, 1 mM MgCl_2_, 2.5 U/ml Benzonase, 1 mM Pefabloc, 0.1 mg/ml lysozyme]. 300 mg glass beads (∅ 0.1 mm) were added and samples were vortexed vigorously at 4 °C for 20 min. 35 µg protein were analyzed on NuPAGE 7% Tris–Acetate SDS-PAGE gels (Invitrogen). Immunodetection was performed with a primary anti-polyhistidine antibody (Qiagen) and a secondary anti-mouse horse reddish peroxidase conjugated antibody (dianova). The blots were developed with the Immobilon Western HRP substrate (Millipore) and chemiluminescence was detected with the ImageQuant LAS4000 imager system (GE Healthcare).

### Large-scale protein production and membrane preparation

For the large-scale protein production, cells of the *P. stutzeri* wild-type strain carrying the expression vector were inoculated 1:50 into 2 l Asn medium supplemented with 34 µg/ml Chloramphenicol in a 5-l baffled flask. The culture was incubated at 32 °C with shaking at 160 rpm. Expression of the protein was induced with 0.02% (w/v) l-arabinose (final concentration) when the OD_600_ reached 0.5–0.6. Cells were harvested 4 h post-induction by centrifugation at 10,000×*g* and 4 °C for 15 min and subsequently flash frozen in liquid nitrogen and stored at −80 °C.

Cell pellets were resuspended in ice-cold high-salt buffer (20 mM Tris pH 8.0, 500 mM NaCl, 1 mM MgCl_2_, 0.1 mg/ml Pefabloc and 2.5 U/ml Benzonase) in a ratio of 3 ml buffer per 1 g cells. Cells were passed through a French pressure cell at 19,000 psi three times. After centrifugation at 20,000×*g* and 4 °C for 10 min to remove unlysed cells and cell debris, membranes were sedimented by centrifugation at 200,000×*g* and 4 °C for 2 h. Subsequently, membranes were washed in ice-cold low-salt buffer (20 mM Tris pH 8.0, 50 mM NaCl) followed by another centrifugation step as described above. Finally, membranes were resuspended in ice-cold binding buffer [20 mM Tris pH 8.0, 50 mM NaCl, 10 mM imidazole, 5% (v/v) glycerol] to a final concentration of 10 mg total protein/ml. The total protein concentration in the membrane was estimated by the BCA assay (Pierce).

### Purification of STM2913

All steps of membrane solubilization and affinity purification were performed at 4 °C. Membranes from 6 l culture were mixed in a ratio of 1:1 with the solubilization buffer [20 mM Tris pH 8.0, 50 mM NaCl, 10 mM imidazole, 5% (v/v) glycerol, 2% (w/v) *n*-dodecyl-β-d-maltoside (DDM)] and incubated with stirring for 30 min. Insoluble particles were removed by centrifugation at 200,000×*g* for 1 h. The supernatant containing solubilized membrane proteins were mixed with 1.5 ml Ni–NTA resin (Qiagen), which were pre-equilibrated with solubilization buffer with a decreased DDM concentration [0.02% (w/v)]. The proteins and matrix were incubated with gentle shaking overnight prior to loading onto a 10-ml gravity column. After washing with 10 column volumes (cv) of washing-buffer [20 mM Tris pH 8.0, 50 mM NaCl, 5% (v/v) glycerol, 0.02% (w/v) DDM], the His_10_-tagged proteins were eluted with a stepwise imidazole gradient of 20, 50 (10 cv) and 250 (5 cv) mM imidazole in washing buffer. Size exclusion chromatography was carried out on a Superdex 200 column connected to an Äkta system. 20 µl of the elution fractions were analyzed by SDS–polyacrylamide gel electrophoresis using 4–12% Bis–Tris gradient gels (Invitrogen). Immunodetection was performed using the PentaHis polyhistidine antibody (Qiagen) according to the manufacturer’s instructions.

## Additional files

**Additional file 1: Figure S2.** Representative dot blot to analyzed production levels of test proteins. Cells were lysed and a fraction of the cell lysate was spotted on the membrane. Target proteins were detected by a poly histidine antibody conjugated to alkaline phosphatase. Defined amounts of poly histidine tagged GFP were loaded as a standard to estimate production levels. A signal ≥ 50 ng corresponds to a yield of ≥ 0.1 mg/l production culture. Samples were collected 2 and 4 h after induction. An uninduced sample (0 h) was used as negative control.

**Additional file 2: Figure S3.**Construct design for the test productions and the GFP folding assay. The constructs were cloned by InFusion cloning. The primers are listed in Additional file 6: Table S1. A All tested proteins were cloned with a C-terminal His_10_-tag. B Selected target proteins were cloned with an N-terminal His_10_-tag. C Selected target proteins were fused with GFP to monitor their folding in *E coli* and *P. stutzeri.*

**Additional file 3: Table S3.** Production level of all tested constructs at different conditions.

**Additional file 4: Figure S1.** Number of constructs scored according to production level at different inducer concentrations and time points. Samples were collected A 2 and B 4 hours after induction. Whole cell lysate was analyzed by dot blot and compared to a GFP-His standard. A poly histidine antibody was used for detection. High: ≥ 0.1 mg/l, low: < 0.1 mg/l, n.d.: not detected.

**Additional file 5: Table S2.** Production levels of target proteins in *Escherichia coli* and *Pseudomonas stutzeri.*

**Additional file 6: Table S1.** List of oligonucleotides used in the study.
