# Supplementary figures and images for: Pseudomonas stutzeri as an alternative host for membrane proteins
Source: Microb Cell Fact. 2017 Sep 20;16:157. doi: 10.1186/s12934-017-0771-0 (PMC5607611; doi:10.1186/s12934-017-0771-0)

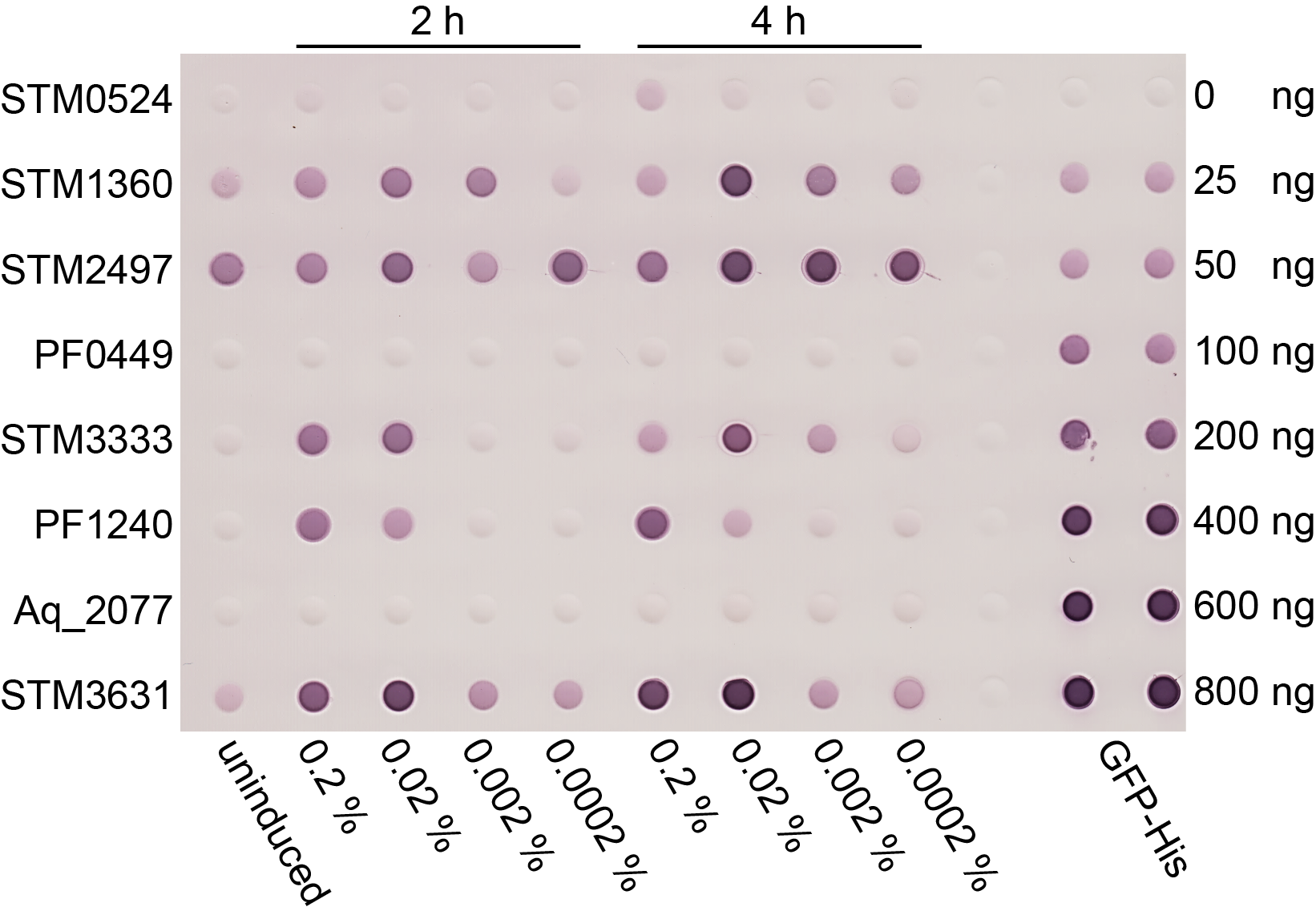

Supplement: Supplementary file 1 — Additional file 1: Figure S2. Representative dot blot to analyzed production levels of test proteins. Cells were lysed and a fraction of the cell lysate was spotted on the membrane. Target proteins were detected by a poly histidine antibody conjugated to alkaline phosphatase. Defined amounts of poly histidine tagged GFP were loaded as a standard to estimate production levels. A signal ≥ 50 ng corresponds to a yield of ≥ 0.1 mg/l production culture. Samples were collected 2 and 4 h after induction. An uninduced sample (0 h) was used as negative control. [file 12934_2017_771_MOESM1_ESM.png]

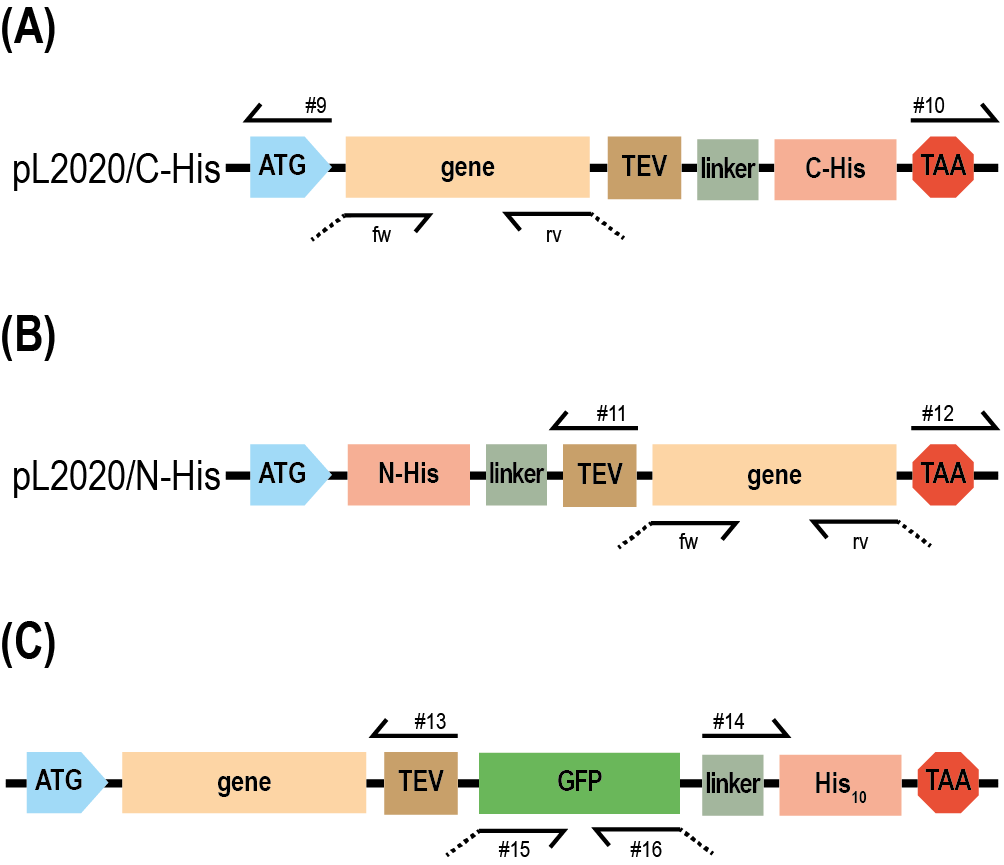

Supplement: Supplementary file 2 — Additional file 2: Figure S3.Construct design for the test productions and the GFP folding assay. The constructs were cloned by InFusion cloning. The primers are listed in Additional file 6: Table S1. A All tested proteins were cloned with a C-terminal His10-tag. B Selected target proteins were cloned with an N-terminal His10-tag. C Selected target proteins were fused with GFP to monitor their folding in E coli and P. stutzeri. [file 12934_2017_771_MOESM2_ESM.png]

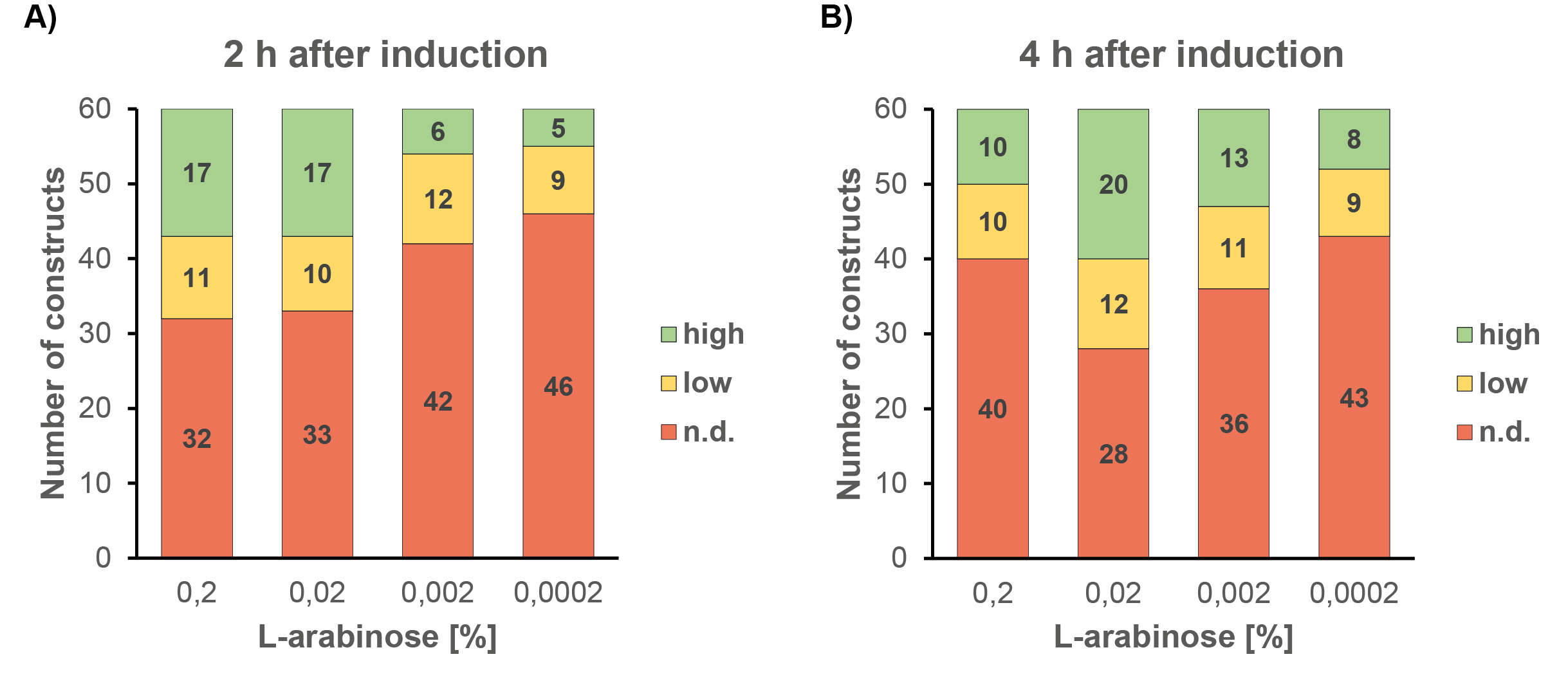

Supplement: Supplementary file 4 — Additional file 4: Figure S1. Number of constructs scored according to production level at different inducer concentrations and time points. Samples were collected A 2 and B 4 hours after induction. Whole cell lysate was analyzed by dot blot and compared to a GFP-His standard. A poly histidine antibody was used for detection. High: ≥ 0.1 mg/l, low: < 0.1 mg/l, n.d.: not detected. [file 12934_2017_771_MOESM4_ESM.png]
