# Supplementary material for: Pseudomonas stutzeri as an alternative host for membrane proteins
Source: Microb Cell Fact. 2017 Sep 20;16:157. doi: 10.1186/s12934-017-0771-0 (PMC5607611; doi:10.1186/s12934-017-0771-0)
Supplement: Supplementary file 3 — Additional file 3: Table S3. Production level of all tested constructs at different conditions. [file 12934_2017_771_MOESM3_ESM.docx]

| **Additional file 3: Table S3.** Production level of all tested constructs at different conditions. | | | | | | | | |
| --- | --- | --- | --- | --- | --- | --- | --- | --- |
| Protein | 2 h | | | | 4 h | | | |
|  | 0.2 | 0.02 | 0.002 | 0.0002 | 0.2 | 0.02 | 0.002 | 0.0002 |
| PA1247-CHis |  |  |  |  |  |  |  |  |
| PA1247-NHis |  |  |  |  |  |  |  |  |
| Aq_1392-CHis |  |  |  |  |  |  |  |  |
| PF0449-CHis |  |  |  |  |  |  |  |  |
| STM006-CHis |  |  |  |  |  |  |  |  |
| 0PF0514-CHis |  |  |  |  |  |  |  |  |
| STM0700-CHis |  |  |  |  |  |  |  |  |
| STM0969-CHis |  |  |  |  |  |  |  |  |
| STM0969-NHis |  |  |  |  |  |  |  |  |
| STM1477-CHis |  |  |  |  |  |  |  |  |
| STM1477-NHis |  |  |  |  |  |  |  |  |
| STM2200-CHis |  |  |  |  |  |  |  |  |
| STM2357-CHis |  |  |  |  |  |  |  |  |
| STM2357-NHis |  |  |  |  |  |  |  |  |
| STM3225-CHis |  |  |  |  |  |  |  |  |
| Aq_1330-CHis |  |  |  |  |  |  |  |  |
| Aq_031-CHis |  |  |  |  |  |  |  |  |
| Aq_031-NHis |  |  |  |  |  |  |  |  |
| STM3166-CHis |  |  |  |  |  |  |  |  |
| STM3356-CHis |  |  |  |  |  |  |  |  |
| STM0832-CHis |  |  |  |  |  |  |  |  |
| STM0832-NHis |  |  |  |  |  |  |  |  |
| STM3765-CHis |  |  |  |  |  |  |  |  |
| STM3765-NHis |  |  |  |  |  |  |  |  |
| STM3746-CHis |  |  |  |  |  |  |  |  |
| STM3746-NHis |  |  |  |  |  |  |  |  |
| STM2913-CHis |  |  |  |  |  |  |  |  |
| STM3512-CHis |  |  |  |  |  |  |  |  |
| STM3512-NHis |  |  |  |  |  |  |  |  |
| STM3541-CHis |  |  |  |  |  |  |  |  |
| STM3541-NHis |  |  |  |  |  |  |  |  |
| STM3801-CHis |  |  |  |  |  |  |  |  |
| STM3801-NHis |  |  |  |  |  |  |  |  |
| STM4482-CHis |  |  |  |  |  |  |  |  |
| PA3553-CHis |  |  |  |  |  |  |  |  |
| PF0520-CHis |  |  |  |  |  |  |  |  |
| PF0520-NHis |  |  |  |  |  |  |  |  |
| STM1360-CHis |  |  |  |  |  |  |  |  |
| PA1236-CHis |  |  |  |  |  |  |  |  |
| PA1236-NHis |  |  |  |  |  |  |  |  |
| PA1569-CHis |  |  |  |  |  |  |  |  |
| Aq_851-CHis |  |  |  |  |  |  |  |  |
| PF2036-CHis |  |  |  |  |  |  |  |  |
| PA2241-CHis |  |  |  |  |  |  |  |  |
| STM0522-CHis |  |  |  |  |  |  |  |  |
| STM0522-NHis |  |  |  |  |  |  |  |  |
| STM3333-CHis |  |  |  |  |  |  |  |  |
| PF0852-CHis |  |  |  |  |  |  |  |  |
| PF1240-CHis |  |  |  |  |  |  |  |  |
| STM0524-CHis |  |  |  |  |  |  |  |  |
| STM0524-NHis |  |  |  |  |  |  |  |  |
| STM2497-CHis |  |  |  |  |  |  |  |  |
| STM3631-CHis |  |  |  |  |  |  |  |  |
| Aq_2077-CHis |  |  |  |  |  |  |  |  |
| PA2760-CHis |  |  |  |  |  |  |  |  |
| PA1436-CHis |  |  |  |  |  |  |  |  |
| PA2495-CHis |  |  |  |  |  |  |  |  |
| Aq_1504-CHis |  |  |  |  |  |  |  |  |
| Aq_1504-NHis |  |  |  |  |  |  |  |  |
| STM3986-CHis |  |  |  |  |  |  |  |  |
| 44 membrane proteins from different transporter families were tested for their production in *Pseudomonas stutzeri* with a C-terminal His_10_ tag. In order to improve their production 16 proteins were additionally cloned with an N-terminal His_10_ tag. Production levels of all 60 tested constructs are shown. Expression was induced with 0.2, 0.02, 0.002 and 0.0002 %[w/v] L-arabinose and samples were collected 2 and 4 h after induction.  **Orange, ≥ 0.1 mg/L; Purple, < 0.1 mg/L; Red, not detected** | | | | | | | | |
