# Supplementary material for: Pseudomonas stutzeri as an alternative host for membrane proteins
Source: Microb Cell Fact. 2017 Sep 20;16:157. doi: 10.1186/s12934-017-0771-0 (PMC5607611; doi:10.1186/s12934-017-0771-0)
Supplement: Supplementary file 5 — Additional file 5: Table S2. Production levels of target proteins in Escherichia coli and Pseudomonas stutzeri. [file 12934_2017_771_MOESM5_ESM.docx]

| **Additional file 5: Table S2.** Production levels of target proteins in *Escherichia coli* and *Pseudomonas stutzeri.* | | | | | | | | |
| --- | --- | --- | --- | --- | --- | --- | --- | --- |
| **Protein** | ***E. coli*** | | | | | | ***P. stutzeri*** | |
|  | pTTQ18-A | pTTQ18-C | pBAD-A | pBAD-C | pQE-A | pQE-C | C-His | N-His |
| Aq_1392 | + | □ | □ | □ | □ | □ | □ | NT |
| PF0449 | □ | □ | □ | + | □ | + | □ | NT |
| STM006 | □ | □ | □ | □ | □ | □ | □ | NT |
| PF0514 | □ | □ | □ | □ | □ | □ | □ | NT |
| STM0700 | ++ | □ | ++ | + | + | □ | ++ | NT |
| STM0969 | ++ | □ | ++ | + | + | □ | + | ++ |
| STM1477 | + | + | + | □ | □ | □ | + | + |
| STM2200 | ++ | ++ | ++ | □ | ++ | ++ | ++ | NT |
| STM2357 | □ | □ | □ | □ | □ | □ | + | ++ |
| STM3225 | □ | ++ | + | ++ | □ | ++ | ++ | NT |
| Aq_1330 | + | □ | □ | □ | □ | □ | ++ | NT |
| Aq_031 | + | □ | ++ | □ | □ | □ | □ | + |
| STM3166 | □ | □ | □ | □ | □ | □ | □ | NT |
| STM3356 | CF | + | □ | □ | □ | □ | □ | NT |
| STM0832 | ++ | ++ | □ | ++ | + | ++ | + | ++ |
| STM3765 | ++ | ++ | □ | □ | □ | + | + | ++ |
| STM3746 | □ | □ | ++ | □ | □ | □ | + | ++ |
| STM2913 | + | + | □ | □ | + | + | ++ | NT |
| STM3512 | + | □ | □ | □ | □ | + | + | ++ |
| STM3541 | + | □ | □ | □ | + | + | + | + |
| STM3801 | + | □ | □ | □ | □ | + | + | ++ |
| STM4482 | + | □ | □ | □ | □ | + | □ | NT |
| Aq_1229 | ++ | + | ++ | ++ | □ | ++ | CF | CF |
| PF0520 | □ | □ | □ | □ | □ | □ | □ | + |
| STM1360 | ++ | ++ | ++ | ++ | ++ | ++ | ++ | NT |
| Aq_851 | □ | □ | ++ | □ | □ | ++ | ++ | NT |
| PF2036 | + | ++ | ++ | ++ | + | + | ++ | NT |
| STM0522 | □ | □ | + | □ | ++ | ++ | □ | □ |
| STM3333 | □ | □ | □ | □ | □ | □ | ++ | NT |
| PF0852 | □ | □ | □ | □ | □ | □ | □ | NT |
| PF1240 | □ | □ | + | □ | □ | ++ | ++ | NT |
| STM0524 | □ | □ | □ | □ | □ | □ | + | □ |
| STM2497 | □ | □ | □ | □ | ++ | □ | ++ | NT |
| STM3631 | □ | □ | + | CF | □ | ++ | ++ | NT |
| Aq_2077 | + | □ | ++ | NT | CF | CF | □ | NT |
| Aq_1504 | + | □ | □ | □ | + | □ | + | □ |
| STM3986 | + | + | + | CF | CF | □ | ++ | NT |
| Production of 37 secondary active transporters was tested in *E. coli* (Surade et al. 2006) and *P. stutzeri* (this study). Scoring in both studies was as follows: ++ ≥ 0.1 µg/ml; + ˂ 0.1µg/ml; □ no protein detected. CF: cloning failed, NT: not tested.  Aq: *Aquifex aeolicus*, STM: *Salmonella enterica*, PF: *Pyrococcus furiosus* | | | | | | | | |
