# Supplementary material for: Pseudomonas stutzeri as an alternative host for membrane proteins
Source: Microb Cell Fact. 2017 Sep 20;16:157. doi: 10.1186/s12934-017-0771-0 (PMC5607611; doi:10.1186/s12934-017-0771-0)
Supplement: Supplementary file 6 — Additional file 6: Table S1. List of oligonucleotides used in the study. [file 12934_2017_771_MOESM6_ESM.docx]

| **Additional file 6: Table S1.** List of oligonucleotides used in the study | | |
| --- | --- | --- |
| 1 | fw_open_pBBR1MSC1 | CGTGCCAGCTGCATTAATG |
| 2 | rv_open_pBBR1MSC1 | GGCGGCGAGGCGGCTACAG |
| 3 | IF-fw_reg-pro-ter | AGCCGCCTCGCCGCCCAATTATGACAACTTGACGGC |
| 4 | IF-rv_reg-pro-ter | AATGCAGCTGGCACGGATAAAACGAAAGGCCCAG |
| 5 | fw_open_pL2010 | TAAGCTTGGCTGTTTTGGCG |
| 6 | rv_open_pL2010 | TAATTCCTCCTGTTAGCCC |
| 7 | fw_MCS-2 | GGGCTAACAGGAGGAATTAC |
| 8 | rv_MCS-2 | CGCCAAAACAGCCAAGC |
| 9 | rv_open1_pL2020 | CATATGTAATTCCTCCTGTTAGCCC |
| 10 | fw_open1_pL2020 | GAAAACCTGTACTTTCAAGGTGGCCG |
| 11 | rv_open2_pL2020 | TTGAAAGTACAGGTTTTCGG |
| 12 | fw_open2_pL2020 | TAAGCTTGGCTGTTTTGGCGGATG |
| 13 | rv_TEV-site | TTGAAAGTACAGGTTTTC |
| 14 | fw_epitope-tag | GGTGGCCGTGGTTCC |
| 15 | IF-rv_pL2040_intro-GFP | GGAACCACGGCCACCCTTGTACAGCTCGTCCATG |
| 16 | IF-fw_pL2040_intro-GFP | AACCTGTACTTTCAAGTGAGCAAGGGCGAGGAG |
| 17 | IF-fw_STM2200_pL2040 | GAGGAATTAACCATGGGTTCCAAAACTAAAACC |
| 18 | IF-rv_STM2200-pL2040 | AAAGTACAGGTTTTCCTTTTTAACGCGTTCCGG |
| 19 | IF-fw_STM1360_pL2040 | GAGGAATTAACCATGTCTCAGAACAAGGCTCGC |
| 20 | IF-rv_STM1360_pL2040 | AAAGTACAGGTTTTCCTGGAAATAATGCTCGCC |
| 21 | IF-fw_STM3356_pL2040 | GAGGAATTAACCATGGAACCCATTACCCTTACC |
| 22 | IF-rv_STM3356_pL2040 | AAAGTACAGGTTTTCCGGATGAAAAGGGAAAAG |
| 23 | IF-fw_Aq1330_pL2040 | GAGGAATTAACCATGAAAAAACTCCTTTCCCTTG |
| 24 | IF-rv_Aq1330_pL2040 | AAAGTACAGGTTTTCGCTGTTTACGTACCTATTC |
| 25 | IF-fw_PF0449_pL2040 | GAGGAATTAACCATGAACATTCCAGAAATGCTTGC |
| 26 | IF-rv_PF0449_pL2040 | AAAGTACAGGTTTTCACTCAACATTTTTAAAACCGAG |
| 27 | IF-fw_Aq1392_pL2040 | GAGGAATTAACCATGTTCATTTACGAGAAAGTTTTCT |
| 28 | IF-rv_Aq1392_pL2040 | AAAGTACAGGTTTTCGAGTAAGATCCTAAACACAG |
| 29 | IF-fw_STM0006_pL2040 | GAGGAATTAACCATGCCTGAGTTTTTCAGTTTTATTAAC |
| 30 | IF-rv_STM0006_pL2040 | AAAGTACAGGTTTTCTCGCGCGATGCCGCCTCC |
| 31 | IF-fw_PF0514_pL2040 | GAGGAATTAACCATGTTCTTAAGTGAACAAGTTCTC |
| 32 | IF-rv_PF0514_pL2040 | AAAGTACAGGTTTTCTGGATTCTTCGAGATAAAGTC |
| 33 | IF-fw_STM0700_pL2040 | GAGGAATTAACCATGAGTAAACCTAAATCCAATAAAATGG |
| 34 | IF-rv_STM0700_pL2040 | AAAGTACAGGTTTTCGCCGTGTTTATTTTTCAATTC |
| 35 | IF-fw_PF0852_pL2040 | GAGGAATTAACCATGGGGTGGATTGAAAGGTATTT |
| 36 | IF-rv_PF0852_pL2040 | AAAGTACAGGTTTTCAGCTGTGGCTAAGTAGGC |
| 37 | IF-fw_STM2357_pL2040 | GAGGAATTAACCATGGATACCCTGCAACAATCTC |
| 38 | IF-rv_STM2357_pL2040 | AAAGTACAGGTTTTCTCCCACTGTGTTACGTAATC |
| 39 | IF-fw_STM3166_pL2040 | GAGGAATTAACCATGCATATAAAATATTTATGGATGTG |
| 40 | IF-rv_STM3166_pL2040 | AAAGTACAGGTTTTCATACAACCCTAACACTTTCC |
| 41 | IF-fw_STM3746_pL2040 | GAGGAATTAACCATGTTTGAACTCGATACCTTATCG |
| 42 | IF-rv_STM3746_pL2040 | AAAGTACAGGTTTTCTCCGGCAAACATTGGCAG |
| 43 | IF-fw_STM0969_pL2040 | GAGGAATTAACCATGCCGAATGTGCAGGAAAAAC |
| 44 | IF-rv_STM0969_pL2040 | AAAGTACAGGTTTTCTGTGTTTCTACGCGCCAG |
| 45 | IF-fw_STM1477_pL2040 | GAGGAATTAACCATGGAAAAAAAACTGGGACTGAG |
| 46 | IF-rv_STM1477_pL2040 | AAAGTACAGGTTTTCACCCACCAGCATCCACGT |
| 47 | IF-fw_STM3225_pL2040 | GAGGAATTAACCATGGCTACGCAACGAGCATCA |
| 48 | IF-rv_STM3225_pL2040 | AAAGTACAGGTTTTCGCTACGCAAGGCGTTATTC |
| 49 | IF-fw_STM0832_pL2040 | GAGGAATTAACCATGCCGGGGTCAACACGTAAAC |
| 50 | IF-rv_STM0832_pL2040 | AAAGTACAGGTTTTCTTTTACGTCAACTTGTTTAATTTG |
| 51 | IF-fw_Aq031_pL2040 | GAGGAATTAACCATGAATAAAAACGTATTTCTCGTTTTAG |
| 52 | IF-rv_Aq031_pL2040 | AAAGTACAGGTTTTCACCTCCCACGTACACTGTAAAG |
| 53 | IF-fw_STM3765_pL2040 | GAGGAATTAACCATGGGTTCCACCAGAAAAGGG |
| 54 | IF-rv_STM3765_pL2040 | AAAGTACAGGTTTTCAGCGGCCCGCGCGCGTCG |
| 55 | IF-fw_STM2913_pL2040 | GAGGAATTAACCATGACAAGTTTACCGACCCCAATTATC |
| 56 | IF-rv_STM2913_pL2040 | AAAGTACAGGTTTTCATCCTTAACTACCAGCTTGTC |
| 57 | IF-fw_STM3512_pL2040 | GAGGAATTAACCATGCCATTAGTCATCGTTGCTATCG |
| 58 | IF-rv_STM3512_pL2040 | AAAGTACAGGTTTTCAACCACCATACCCAACAGC |
| 59 | IF-fw_STM3541_pL2040 | GAGGAATTAACCATGAGTACATTAACACTG |
| 60 | IF-rv_STM3541_pL2040 | AAAGTACAGGTTTTCGCTCAGCAGTTGGAAAG |
| 61 | IF-fw_STM3801_pL2040 | GAGGAATTAACCATGCACGCTCAAATCTGGGTAG |
| 62 | IF-rv_STM3801_pL2040 | AAAGTACAGGTTTTCGATGATAAAGGAAAG |
| 63 | IF-fw_STM4482_pL2040 | GAGGAATTAACCATGCCATTAATAATTATTGC |
| 64 | IF-rv_STM4482_pL2040 | AAAGTACAGGTTTTCATGCAGAACCGCATTCAGC |
| 65 | IF-fw_Aq1229_pL2040 | GAGGAATTAACCATGCTTACTAAGGAAGAAAAAAAGG |
| 66 | IF-rv_Aq1229_pL2040 | AAAGTACAGGTTTTCAGTGCTTCTCTTTTTTATTTC |
| 67 | IF-fw_PF0520_pL2040 | GAGGAATTAACCATGGGAAGGTGGAAGACCG |
| 68 | IF-rv_PF0520_pL2040 | AAAGTACAGGTTTTCAGTAGTCTCAACTGGGAGC |
| 69 | IF-fw_Aq851_pL2040 | GAGGAATTAACCATGTTGATAAAATATGAATGTG |
| 70 | IF-rv_Aq851_pL2040 | AAAGTACAGGTTTTCGAGCCATCCCTTCC |
| 71 | IF-fw_PF2036_pL2040 | GAGGAATTAACCATGAACGACATTAAGAAGGTAAC |
| 72 | IF-rv_PF2036_pL2040 | AAAGTACAGGTTTTCTAGCCACCCTTTCCTC |
| 73 | IF-fw_STM0522_pL2040 | GAGGAATTAACCATGGTCGGCGGTTTTTTTATACTG |
| 74 | IF-rv_STM0522_pL2040 | AAAGTACAGGTTTTCACCTGCGAGTTTTTTCTCTC |
| 75 | IF-fw_STM3333_pL2040 | GAGGAATTAACCATGGTGTCGCAGGACAAC |
| 76 | IF-rv_STM3333_pL2040 | AAAGTACAGGTTTTCTTGTTCTGCATGGCTGAC |
| 77 | IF-fw_PF1240_pL2040 | GAGGAATTAACCATGGGTCCTGACATTAAG |
| 78 | IF-rv_PF1240_pL2040 | AAAGTACAGGTTTTCCCTCAACACCTGCTC |
| 79 | IF-fw_STM0524_pL2040 | GAGGAATTAACCATGTTCCCTGTTATTAACC |
| 80 | IF-rv_STM0524_pL2040 | AAAGTACAGGTTTTCTTTAATGCGTTCCCACGG |
| 81 | IF-fw_STM2497_pL2040 | GAGGAATTAACCATGACGCGCCGTGCTATCGGG |
| 82 | IF-rv_STM2497_pL2040 | AAAGTACAGGTTTTCCTGATGCGGGGGCTCC |
| 83 | IF-fw_STM3631_pL2040 | GAGGAATTAACCATGCCGCAACCTGGCCTTAC |
| 84 | IF-rv_STM3631_pL2040 | AAAGTACAGGTTTTCACTTTTGACCCTGGCCTGC |
| 85 | IF-fw_Aq2077_pL2040 | GAGGAATTAACCATGGAAGTTAAAAGGGAACACTG |
| 86 | IF-rv_Aq2077_pL2040 | AAAGTACAGGTTTTCAGCACTCTCATGGTTTCTTC |
| 87 | IF-fw_Aq1504_pL2040 | GAGGAATTAACCATGGTGGTAAAAAAGTTAAACC |
| 88 | IF-rv_Aq1504_pL2040 | AAAGTACAGGTTTTCTACTAAAATCCTTGCCTCCG |
| 89 | IF-fw_STM3986_pL2040 | GAGGAATTAACCATGCATTTTCGCGCCATTACCC |
| 90 | IF-rv_STM3986_pL2040 | AAAGTACAGGTTTTCTTCACGCCAGAAAGTCG |
| 91 | IF-fw_STM0969_pL2030 | AACCTGTACTTTCAACCGAATGTGCAGGAAAAAC |
| 92 | IF-rv_STM0969_pL2030 | AAACAGCCAAGCTTATGTGTTTCTACGCGCCAG |
| 93 | IF-fw_STM0832_pL2030 | AACCTGTACTTTCAACCGGGGTCAACACGTAAAC |
| 94 | IF-rv_STM0832_pL2030 | AAACAGCCAAGCTTATTTTACGTCAACTTGTTTAATTTGC |
| 95 | IF-fw_STM2357_pL2030 | AACCTGTACTTTCAAGATACCCTGCAACAATCTC |
| 96 | IF-rv_STM2357_pL2030 | AAACAGCCAAGCTTATCCCACTGTGTTACGTAATC |
| 97 | IF-fw_STM3541_pL2030 | AACCTGTACTTTCAAAGTACATTAACACTGG |
| 98 | IF-rv_STM3541_pL2030 | AAACAGCCAAGCTTAGCTCAGCAGTTGGAAAGC |
| 99 | IF-fw_PF0520_pL2030 | AACCTGTACTTTCAAGGAAGGTGGAAGACCG |
| 100 | IF-rv_PF0520_pL2030 | AAACAGCCAAGCTTAAGTAGTCTCAACTGGG |
| 101 | IF-fw_Aq031_pL2030 | AACCTGTACTTTCAAAATAAAAACGTATTTCTC |
| 102 | IF-rv_Aq031_pL2030 | AAACAGCCAAGCTTAACCTCCCACGTACAC |
| 103 | IF-fw_PA2241_pL2040 | GAGGAATTAACCATGCTCGGCTCCGCCTTCTG |
| 104 | IF-rv_PA2241_pL2040 | AAAGTACAGGTTTTCATCGGTGCGCCAGGG |
| 105 | IF-fw_PA3553_pL2040 | GAGGAATTAACCATGAAGCCCTATCCGATCGAC |
| 106 | IF-rv_PA3553_pL2040 | AAAGTACAGGTTTTCTGGCGTGGAAGGCTCGCTGG |
| 107 | IF-fw_PA2495_pL2040 | GAGGAATTAACCATGATTCACGCGCAGTCGATCCG |
| 108 | IF-rv_PA2495_pL2040 | AAAGTACAGGTTTTCGGCGCTGGGTTGCCAGCCAC |
| 109 | IF-fw_PA2760_pL2040 | GAGGAATTAACCATGTTGAAGAAAAGGATTTG |
| 110 | IF-rv_PA2760_pL2040 | AAAGTACAGGTTTTCGAACACGCTGAACGGGTACTC |
| 111 | IF-fw_PA1247_pL2040 | GAGGAATTAACCATGACCCGCACGGTGAAACGC |
| 112 | IF-rv_PA1247_pL2040 | AAAGTACAGGTTTTCGTTCTCCGCCAGCGCCAC |
| 113 | IF-fw_PA1436_pL2040 | GAGGAATTAACCATGACGCCGCGCGCCGGGATTTC |
| 114 | IF-rv_PA1436_pL2040 | AAAGTACAGGTTTTCTGTCGCCGTCGCCCCAGC |
| 115 | IF-fw_PA1569_pL2040 | GAGGAATTAACCATGAATCAACGTCCCGGGACG |
| 116 | IF-rv_PA1569_pL2040 | AAAGTACAGGTTTTCGGAGCGGATGGGACGGAAG |
| 117 | IF-fw_PA1236_pL2040 | GAGGAATTAACCATGAGCGCTGACGACAAGG |
| 118 | IF-rv_PA1236_pL2040 | AAAGTACAGGTTTTCGTCCTTCTGGCGCAGCATC |
| 119 | IF-fw_PA1247_pL2030 | AACCTGTACTTTCAAACCCGCACGGTGAAACGC |
| 120 | IF-rv_PA1247_pL2030 | AAACAGCCAAGCTTAGTTCTCCGCCAGCGCCACG |
| 121 | IF-fw_PA1236_pL2030 | AACCTGTACTTTCAAAGCGCTGACGACAAGGTATC |
| 122 | IF-rv_PA1236_pL2030 | AAACAGCCAAGCTTAGTCCTTCTGGCGCAGCATC |
| 123 | IF-fw_STM0522_pL2030 | AACCTGTACTTTCAAGTCGGCGGTTTTTTTATACTG |
| 124 | IF_rv_STM0522_pL2030 | AAACAGCCAAGCTTAACCTGCGAGTTTTTTCTC |
| 125 | IF-fw_Aq1229_pL2030 | AACCTGTACTTTCAACTTACTAAGGAAGAAAAAAAGG |
| 126 | IF-rv_Aq1229_pL2030 | AAACAGCCAAGCTTAAGTGCTTCTCTTTTTTATTTC |
| 127 | IF-fw_STM3765_pL2030 | AACCTGTACTTTCAAGGTTCCACCAGAAAAGG |
| 128 | IF-rv_STM3765_pL2030 | AAACAGCCAAGCTTAAGCGGCCCGCGCGCGTCG |
| 129 | IF-fw_STM3746_pL2030 | AACCTGTACTTTCAATTTGAACTCGATACCTTATCG |
| 130 | IF-rv_STM3746_pL2030 | AAACAGCCAAGCTTATCCGGCAAACATTGGCAG |
| 131 | IF-fw_STM1477_pL2030 | AACCTGTACTTTCAAGAAAAAAAACTGGGACTGAGC |
| 132 | IF-rv_STM1477_pL2030 | AAACAGCCAAGCTTAACCCACCAGCATCCACGTAG |
| 133 | IF-fw_STM3512_pL2030 | AACCTGTACTTTCAACCATTAGTCATCGTTGCTATC |
| 134 | IF-rv_STM3512_pL2030 | AAACAGCCAAGCTTAAACCACCATACCCAACAG |
| 135 | IF-fw_STM3801_pL2030 | AACCTGTACTTTCAACACGCTCAAATCTGGGTAG |
| 136 | IF-rv_STM3801_pL2030 | AAACAGCCAAGCTTAGATGATAAAGGAAAGCAGG |
| 137 | IF-fw_STM0524_pL2030 | AACCTGTACTTTCAATTCCCTGTTATTAACCGC |
| 138 | IF-rv_STM0524_pL2030 | AAACAGCCAAGCTTATTTAATGCGTTCCCACGG |
| 139 | IF-fw_Aq1504_pL2030 | AACCTGTACTTTCAAGTGGTAAAAAAGTTAAACCC |
| 140 | IF-rv_Aq1504_pL2030 | AAACAGCCAAGCTTATACTAAAATCCTTGCCTC |
| Primer names are combinations of the gene’s locus tag and the respective vector the gene was introduced to. IF: InFusion cloning, fw: forward, rv: reverse, MCS: multiple cloning site, reg-pro-ter: regulator-promoter-terminator. | | |
